# Supplementary material for: Octave spanning operation of visible to SWIR integrated coil-stabilized Brillouin lasers
Source: Light Sci Appl. 2026 Jan 2;15:31. doi: 10.1038/s41377-025-02133-0 (PMC12757599; doi:10.1038/s41377-025-02133-0)
Supplement: Supplementary file 1 — Supplementary Information for Octave Spanning Operation of Visible to SWIR Integrated Coil-Stabilized Brillouin Lasers [file 41377_2025_2133_MOESM1_ESM.docx]

**Supplementary Information for Octave Spanning Operation of Visible to SWIR Integrated Coil-Stabilized Brillouin Lasers**

**Meiting Song^1^, Nitesh Chauhan^2, 3^, Mark W. Harrington^1^, Nick Montifiore^1^, Kaikai Liu^1^, Andrew S. Hunter^1^, Chris Caron^4^, Andrei Isichenko^1^, Robert J. Niffenegger^4^, Daniel J. Blumenthal^1,*^**

*^1^Department of Electrical and Computer Engineering, University of California Santa Barbara, Santa Barbara, CA 93106, USA*

*^2^Time and Frequency Division, National Institute of Standards and Technology, Boulder, CO 80305, USA*

*^3^Department of Physics, University of Colorado, Boulder, CO 80309, USA*

*^4^Department of Electrical and Computer Engineering, University of Massachusetts Amherst, Amherst, MA 01003, USA*

** Corresponding author*

**S1. Resonator waveguide design and resonances**

The waveguide structure with the dilute mode design consists of 15 µm SiO_2_ lower cladding, 40 nm Si_3_N_4_ waveguide, and 6 µm SiO_2_ upper cladding (Fig. S1a), with the exception of the coil resonator for 1550 nm, whose waveguide thickness is 80 nm. The thicker waveguide lowers the critical bending radius, which allows the fitting of 4-m cavity length into the area of the chip. The resonance transmission spectrum is calibrated by the fiber MZI FSR. The loaded quality factors (Q) are 42 million for the SBS resonator and 62 million for the coil resonator at 674 nm (Fig. S1b), 36 million for the SBS resonator and 43 million for the coil resonator at 698 nm (Fig. S1c), 28 million for the SBS resonator^1^ and 36 million for the coil resonator at 1550 nm^2^.


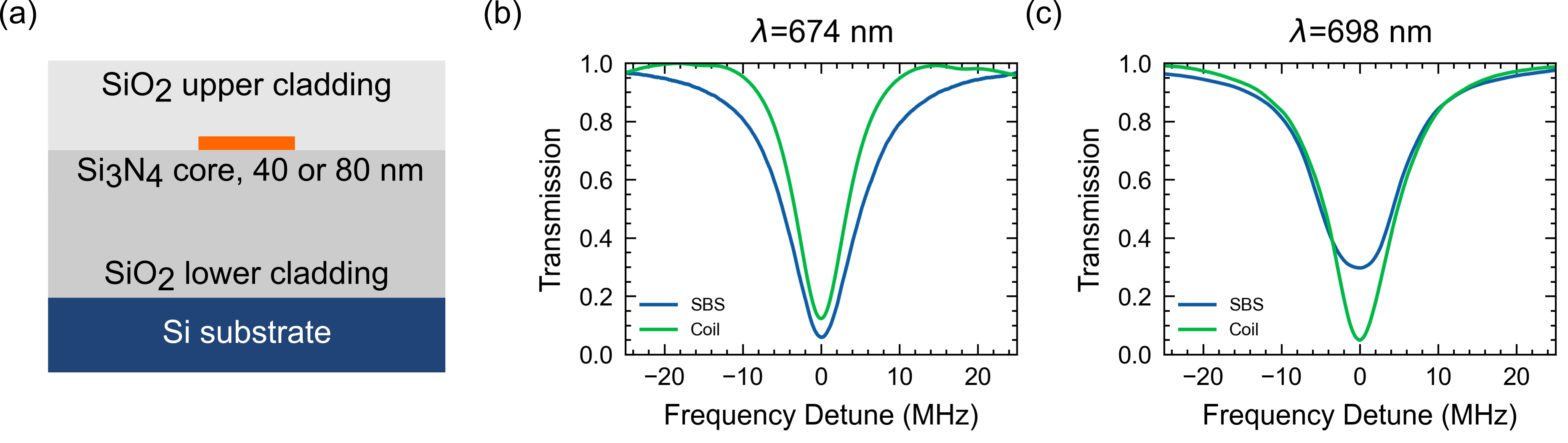


**Fig. S1 Waveguide structure and resonance spectrum.** **a** Waveguide layer stack with 15 µm SiO_2_ lower cladding, 40 nm Si_3_N_4_ waveguide, and 6 µm SiO_2_ upper cladding. **b** Resonance spectrum of SBS resonator (42 million loaded Q) and coil resonator (62 million loaded Q) at 674 nm. **c** Resonance spectrum of SBS resonator (36 million loaded Q) and coil resonator (43 million loaded Q) at 698 nm.

A summary of the reference cavity coil resonators can be found in Table 1 below.

**Table 1 Coil resonator summary**

| Wavelength | Coil length | Q, intrinsic | Q, loaded | Loss | Cavity mode volume |
| --- | --- | --- | --- | --- | --- |
| 674 nm | 3 m | 94 million | 62 million | 0.63 dB m^-1^ | 8.8×10^6^ µm^3^ |
| 698 nm | 3 m | 110 million | 43 million | 0.53 dB m^-1^ | 9.8×10^6^ µm^3^ |
| 1550 nm | 4 m | 41 million | 34 million | 0.61 dB m^-1^ | 39.0×10^6^ µm^3^ |

**S2. SBS threshold measurement**

The SBS lasing threshold is measured fitting the pump-SBS power relation to the theoretical model of SBS lasing^3^. The on-chip pump and S1 powers are estimated from the measured fiber power and the known fiber–chip coupling losses, assuming symmetric losses at both ends of the chip. The relationship between SBS power and pump power is then calculated based on the quality factor (Q) of the resonance and the fundamental linewidth that best match the experimental data. From this model, the SBS lasing threshold power is extracted. The threshold measurements for 674 nm and 698 nm are shown in Fig. S2 and the measurement of 1550 nm can be found in previous publication^1^.

**
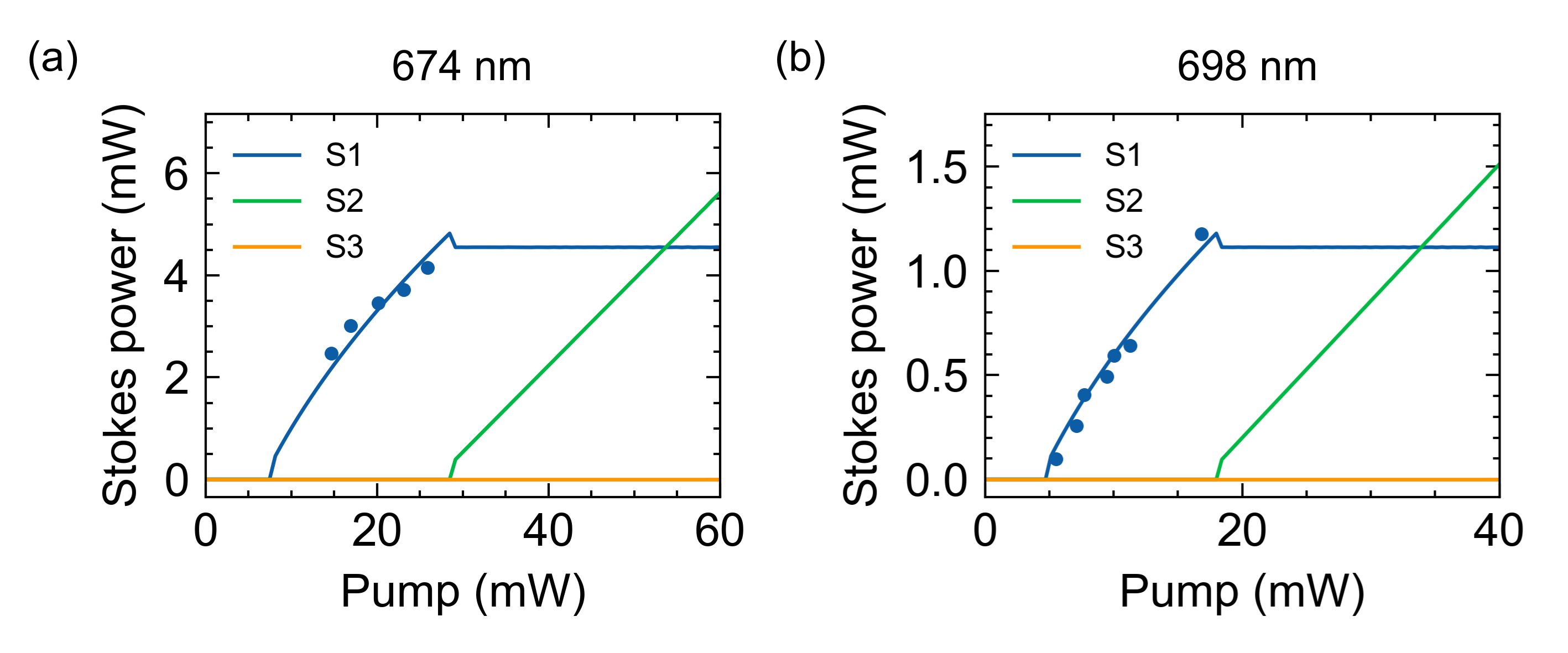
**

**Fig. S2 SBS lasing threshold.** SBS lasing threshold measurement and calculation for **a** 674 nm and **b** 698 nm**.** The solid lines are calculated power of first (S1), second (S2) and third (S3) order Stokes emission based on the theoretical model. The blue dots are measured on-chip pump and S1 power.

**S3. PDH lock system**

The pump laser lock is implemented using an FPGA-based device that serves as the waveform generator, mixer, low-pass filter, and PID controller (Fig. S3). The modulation and servo signals are combined with a bias-tee and applied to the current modulation port of the pump laser. The coil reference lock is implemented with a Vescent laser lock module, which provides the low-pass filtering and PID control. A separate waveform generator supplies the sine wave to drive the EOM and to mix with the photodetector signal. The PID output tunes the AOM frequency via an amplified VCO signal. The modulation frequencies for the two PDH locks are set at least 10 MHz apart to prevent interaction between the loops.


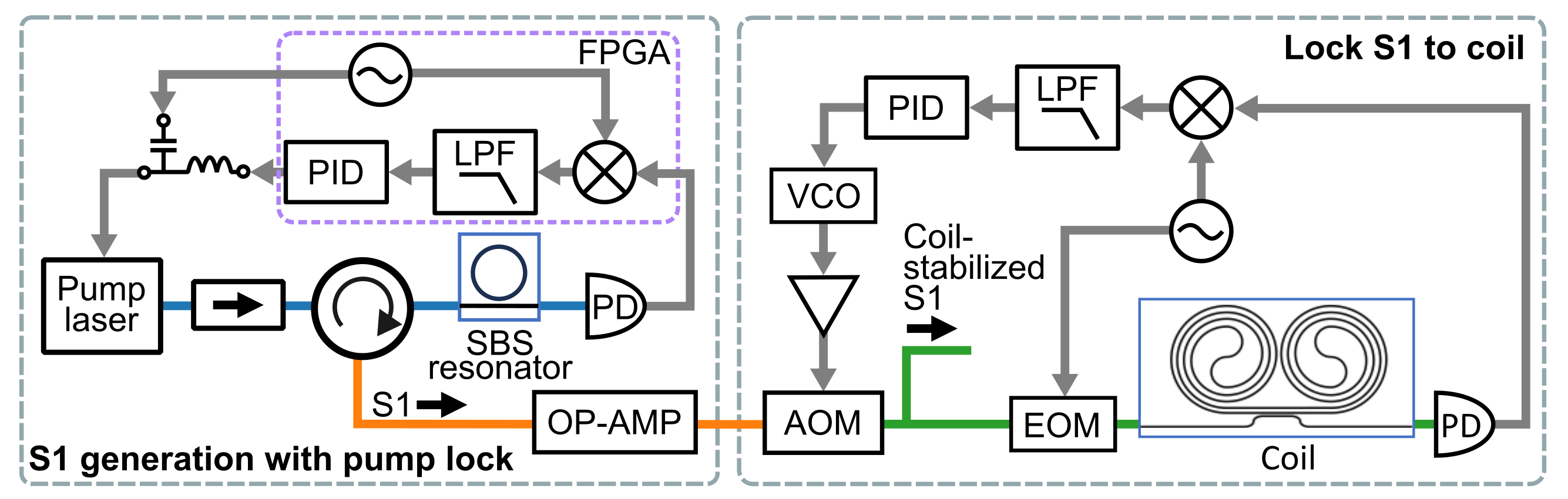


**Fig. S3 PDH laser lock system block diagram.** Pump laser PDH lock is implemented with an FPGA controller and applied via current modulation, while the coil reference PDH lock uses a Vescent module with a waveform generator for driving EOM. The two modulation frequencies are separated by at least 10 MHz to avoid loop interactions.

**S4. Laser frequency noise measurement analysis**

Our laser frequency noise measurements at different offsets are limited by different components. At high frequency offsets >1 MHz, the measurement noise floor can be limited by the photodetector (PD) bandwidth. We convert the power spectral density of the photodetector dark noise to frequency noise considering the detected power and the FSR of the fiber MZI noise measurement (red dashed curves in Fig. S4). In the figure, the specific PD noise limit shown is that of the coil-stabilized SBS laser measurement, as it may vary with the level of voltage output that depends on different detected power and PD gain settings for other measurements. The frequency noise of the laser decreases with a higher offset until it reaches the white noise floor. The fundamental linewidth $\Delta\nu_{f}$ is defined as

$$\Delta\nu_{f}=\pi S_{w}$$

where $S_{w}$ is the white noise floor. The frequency noise above 1 MHz is increased due to the added noise of the amplifier. The added noise of the injection-locked amplifier used at 674 nm and 698 nm can be reduced by improving the locking between the seed and high-power lasers. This can be achieved through better alignment to the high-power laser diode, increased seed laser (S1) power, and the use of a single-mode laser diode as the high-power laser.

In the mid-frequency offsets around 1 kHz – 1 MHz, the laser frequency noise is determined by the PDH locking performance. A higher loop bandwidth of the servo feedback loop can push the “servo bump” into a higher frequency offset and lower the integral linewidth of the laser. In the low frequency offset range <1 kHz, the stability of the fiber MZI is affected by acoustic waves and vibrations in the environment, which limits the OFD measurement noise floor (yellow curves in Fig. S4). Therefore, we use our stabilized laser system (SLS), which is a stabilized source down to 1 Hz offset, as a reference to measure the coil-locked SBS laser noise (light blue curves in Fig. S4). We mix the coil-locked SBS laser with a frequency comb that is locked to the SLS. Then we measure the beat note signal with a frequency counter, which is locked to a Rb reference, and convert the result to frequency noise. The frequency noise of the pump and the standalone SBS laser are not limited by the fiber MZI noise, so they are only measured with OFD. The 1/π reverse integral linewidth $\Delta\nu_{i}$ is then defined as the frequency to which the phase noise integral from the high-frequency offset is 1/π

$$\int_{\Delta\nu_{i}}^{\infty} S_{\phi}\left( f \right)df=\int_{\Delta\nu_{i}}^{\infty} \frac{S_{\nu}\left( f \right)}{f^{2}}df=\frac{1}{\pi}$$

where $S_{\phi}$ is the single-sideband phase noise and $S_{\nu}$ is the single-sideband frequency noise. The high-frequency limit of the integration is 1 MHz, which is close to the upper limit of the OFD frequency noise measurement system.


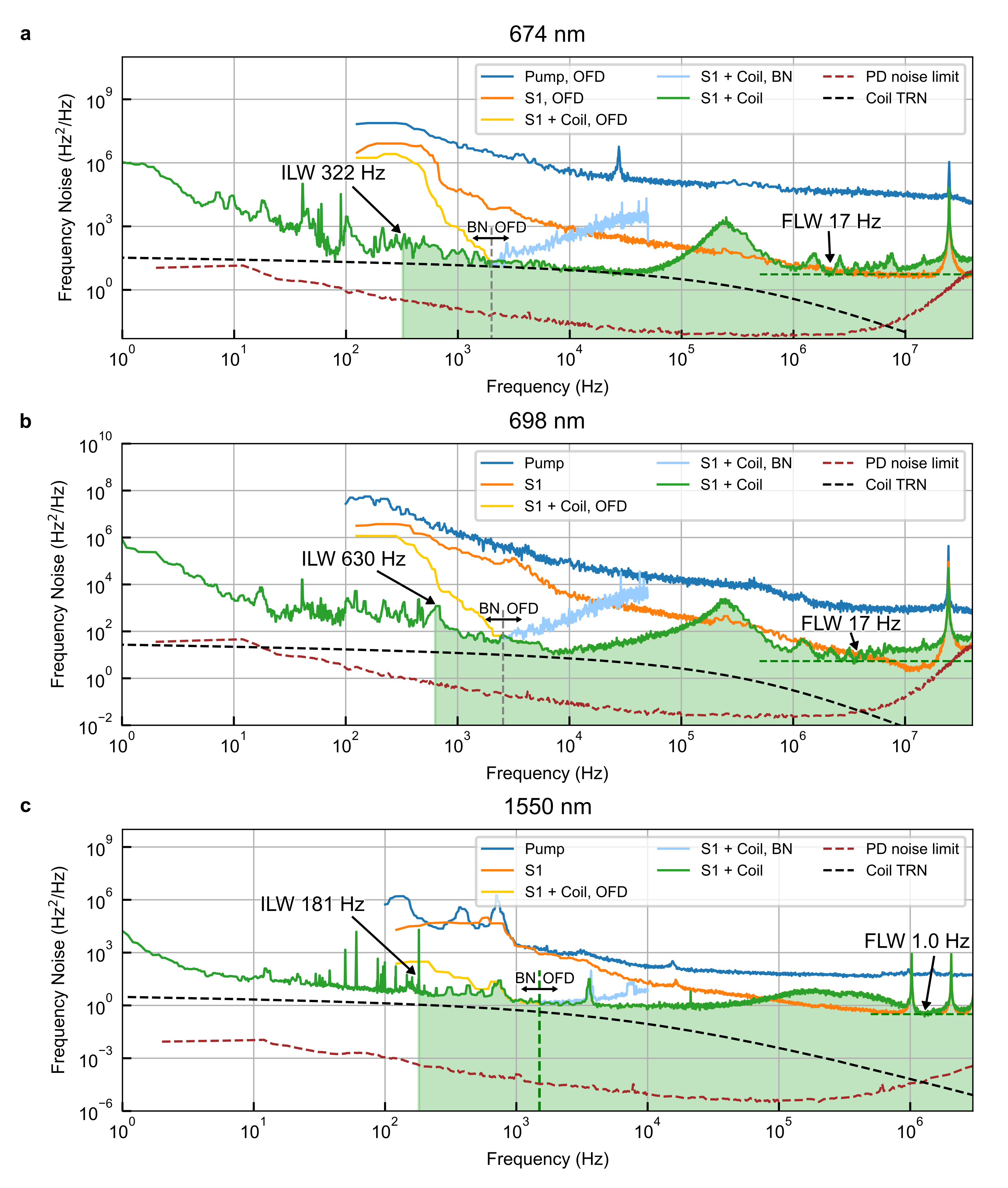


**Fig. S4 Frequency noise of the integrated coil-stabilized Brillouin lasers.** Frequency noise with photodetector (PD) noise limits at **a** 674 nm, **b** 698 nm, and **c** 1550 nm. The green horizontal dashed line shows the fundamental linewidth of the coil-stabilized SBS laser. The green shaded area shows the integral region of the integral linewidth calculation. The grey vertical dashed line shows the stitching between OFD and beat note (BN) measurements. The yellow and light blue curves are OFD and BN data before the stitch. The red dashed line shows the PD noise limit.

**References**

1. Gundavarapu, S. *et al.* Sub-hertz fundamental linewidth photonic integrated Brillouin laser. *Nature Photon* **13**, 60–67 (2019).

2. Sun, S. *et al.* Integrated optical frequency division for microwave and mmWave generation. *Nature* **627**, 540–545 (2024).

3. Behunin, R. O., Otterstrom, N. T., Rakich, P. T., Gundavarapu, S. & Blumenthal, D. J. Fundamental noise dynamics in cascaded-order Brillouin lasers. *Phys. Rev. A* **98**, 023832 (2018).
